# Identification of an LPS-Induced Chemo-Attractive Peptide from *Ciona robusta*

**DOI:** 10.3390/md18040209

**Published:** 2020-04-12

**Authors:** Valeria Longo, Alessandra Longo, Annamaria Martorana, Antonino Lauria, Giuseppa Augello, Antonina Azzolina, Melchiorre Cervello, Paolo Colombo

**Affiliations:** 1 Institute for Biomedical Research and Innovation, National Research Council, Via Ugo La Malfa 153, 90146 Palermo, Italy; valeria.longo@irib.cnr.it (V.L.); alessandra.longo@irib.cnr.it (A.L.); giuseppa.augello@irib.cnr.it (G.A.); antonina.azzolina@irib.cnr.it (A.A.); melchiorre.cervello@irib.cnr.it (M.C.); 2 Dipartimento di Scienze e Tecnologie Biologiche Chimiche e Farmaceutiche “STEBICEF”, University of Palermo, Viale delle Scienze, 90128 Palermo, Italy; annamaria.martorana@unipa.it (A.M.); antonino.lauria@unipa.it (A.L.)

**Keywords:** *Ciona robusta*, inflammation, chemoattractive peptide, NF-kB, 3D modelling

## Abstract

Background: Previously published work has demonstrated that the LPS injection of *Ciona robusta* leads to the overexpression of a truncated form of an immune-related mRNA (C8short) by means of *Ciona robusta* (CR) alternative polyadenylation (APA) (CR-APA). Methods: The 3D structure of the C8short-derived *Ciona robusta* chemo-attractive peptide (CrCP) was evaluated by homology modeling. The biological activity of the CrCP was studied in vitro using a primary human dermal cell line (HuDe). Real-Time PCR was used to investigate the expression levels of genes involved in cell motility. NF-κB signaling was studied by western blotting. Results: In silico modeling showed that CrCP displayed structural characteristics already reported for a short domain of the vertebrate CRK gene, suggesting its possible involvement in cell migration mechanisms. In vitro assays demonstrated that CrCP was capable of inducing the motility of HuDe cells in both wound healing and chemo-attractive experiments. qPCR demonstrated the capability of CrCP to modulate the expression of the matrix metalloproteinase-7 (MMP-7) and E-cadherin genes. Finally, western blot analysis demonstrated that treatment with CrCP induced activation of the NF-κB signaling pathway. Conclusion: Our results describe the characterization of the 3D structure and chemo-attractive activity of an LPS-induced CrCP peptide from *Ciona robusta*.

## 1. Introduction

Tunicates are the closest relatives to vertebrates [1,2] and, like other invertebrates, their immune response relies exclusively on innate immunity. In this respect, ascidians are one of the most studied biological systems due to their phylogenetic position, which makes them a particularly useful model for studying the evolutionary events which occurred during the invertebrate–vertebrate transition, leading to the appearance of adaptive immunity characterized by receptor diversification through somatic recombination [3]. Furthermore, the ascidian *Ciona robusta* (formerly *Ciona intestinalis* type A) [4] was the first invertebrate chordate to have its genome sequenced, allowing for the analysis of developmental mechanisms, genome-wide gene regulatory networks, epigenetic regulatory mechanisms and gene expression profiles at single-cell resolution [5,6,7,8]. In this scenario, *Ciona robusta* has been extensively utilized to study inflammatory response after challenge with foreign agents, such as the injection of erythrocytes [9,10], proteins [11] and LPS [12] into the tunic and, in particular, into the pharynx, which is considered the immunocompetent organ [13]. These stimuli can induce a complex cascade of events, leading to the activation of immune-related gene pathways and to the recruitment of numerous hemocytes infiltrating the tunic tissue, where they can release several inflammation mediators [14]. At a molecular level, it has been demonstrated that the pharynx responds to these different stimuli through the activation of an alternative complementary pathway [15], the increase in phenoloxidase system activities [16,17], the increased production of C-type lectins (CiMBL) [18], galectins [19,20], cytokines [21,22] (CiTNFα, IL17-like), phenoloxidase [23], FACIT-type IX-like collagen [24] and the increased transcription of an activator gene (CiCAP) [25]. These studies not only made the identification of distinct immune-related gene families possible, but also allowed researchers to shed some light on the evolution of several mechanisms which generate sequence diversity in genes with immune functions [26]. Moreover, within a few days post-LPS inoculum, it is possible to observe a tunic repair response with the formation of a capsule at the injection site, followed by a wound repair response represented by a thick capsule [9,10]. These events involve the hemocyte infiltration of various cell types [27], hemocyte and epidermis activities, vacuolization and cell disruption, while cell products can contribute to forming the capsule components and/or cause a tunic wound [28].

In addition, our research group has demonstrated that, during LPS-induced inflammatory response, *Ciona robusta* can activate additional mechanisms regulating the expression of immune-related genes, such as the activation of alternative polyadenylation (APA) sites that can generate novel forms of mRNAs with different levels of expression and tissue localization [29]. In particular, our group described the characterization of a transcript (C8long) containing protein domains with relevant homologies with several components of the Receptor Transporting Protein (RTP) family which are involved in modulating G protein-coupled receptors trafficking and function [30]. In our previous work, we demonstrated that C8long expression was not modulated by the injection of LPS, and in situ hybridization demonstrated that this transcript is mainly expressed in compartment/morula and signet ring cells located in a tightly packed cluster within the vessel lumen [29]. However, after LPS stimulation, we observed the activation of an APA site within the first intron of the C8long gene, leading to the upregulation of a shorter transcript lacking the RTP elements (C8short) [29]. Furthermore, we demonstrated that this shorter mRNA is preferentially accumulated in a large part of the vessels, densely populated with hemocytes and endothelial cells, which appeared to be marked in various regions of the pharynx bars. This finding demonstrated that the activation of the APA site could modulate the expression and tissue localization of the C8short transcript [29]. However, besides its temporal expression and tissue localization, homology sequence analysis did not suggest a specific functional role for the C8short-derived peptide. 

In this paper, we studied the biological function of the synthetic C8short-derived *Ciona robusta* chemo-attractive peptide (CrCP). We examined its 3D structure by means of in silico modeling, which showed a certain degree of homology to a protein domain of the human cancer-related signaling adaptor protein CT10 Regulator of Kinase (CRK) [31], a vertebrate gene that has been demonstrated to induce cytoskeletal reorganization during cell migration. Furthermore, using a primary dermal human cell line (HuDe) as a test system, we were able to show that CrCP displays the ability to work as a chemo-attractive peptide.

## 2. Results

### 2.1. CrCP Structure and Homology

**Figure 1:**
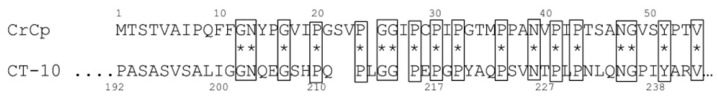
Alignment of the *Ciona robusta* chemo-attractive peptide (CrCP) amino acid sequence and the fragment of the top-scoring protein, CT-10-Regulated Kinase II. Numbers indicate the relative position of the amino acids in the full-length sequences.

To the best of our knowledge, CrCP’s crystal structure is not fixed; thus, with the aim of finding homologous proteins from which to hypothesize its biological functions, molecular modeling protocols were applied. The primary amino acid sequence, retrieved from our previous work [29], was submitted to homology modeling analysis. The top-scoring homologue protein was the CT10-Regulated Kinase isoform II (PDB ID: 2EYZ) [31]. Our analysis showed 40.5% identity (54.8% similarity) with a 42 amino acid (aa) overlap. In particular, the homology that appeared to be the most frequent amino acid was proline, which is usually significantly present in so-called Intrinsically Disordered Proteins (IDPs) (Figure 1) [32]. The chosen model was optimized by adding hydrogen atoms and charges, and by minimizing the structure using Schrödinger’s Prime refinement tool. 

**Figure 2:**
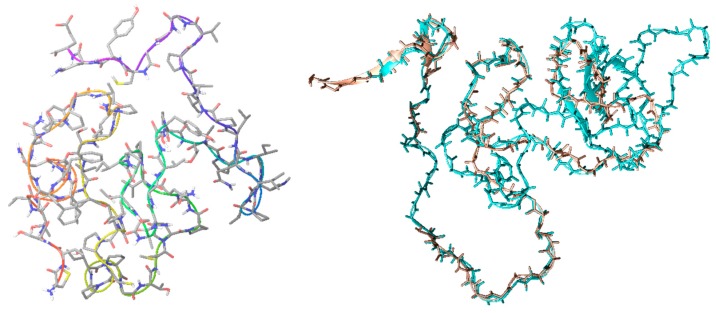
**Left**: 3D structure of CrCP built by homology modeling and optimized; **right**: superposition of CT10 (PDB ID: 2EYZ) fragment (cyan), used for the homology process, and CrCP (brown).

Finally, the model was submitted to a 100 ns run of molecular dynamics, as described in the Materials and Methods section. The results obtained (Figure 2, left) showed that the peptide structure reaches convergence after 30 ns, confirming the structural behavior of IDPs. The corresponding portion of CT10, used in the homology protocol, shows the same 3D structural characteristic (Figure 2, right).

**Figure 3:**
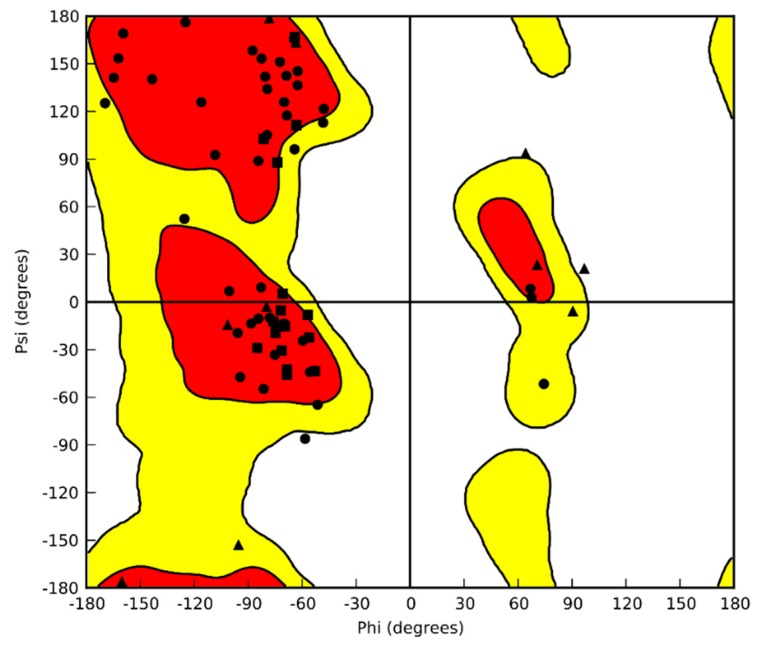
Ramachandran plot of CrCP.

Moreover, the examination of Ramachandran plot (Figure 3) provided further evidence of the model’s strength, since almost all residues fall in the allowed regions of the plot.

### 2.2. Cytotoxic Effects of CrCP on HuDe Cell Line Viability

**Figure 4:**
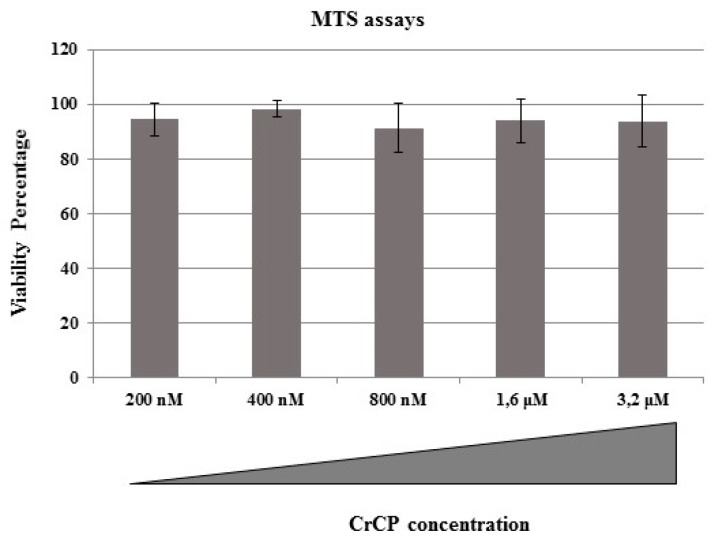
Cell viability assay: MTS assay shows that increasing concentrations of CrCP did not impair the viability of human dermal cell line (HuDe).

In a previously published paper, we demonstrated that CrCP did not show any cytotoxic or hemolytic effects on either immortalized or primary human cell lines within a large range of concentrations and up to 48 h of incubation [33]. In the current study, we decided to evaluate the effect of CrCP on the viability of HuDe cells by MTS assay. The HuDe cells were incubated with increasing concentrations of CrCP (200 nM, 400 nM, 800 nM, 1.6 μM and 3.2 μM) for 24 h. Three independent experiments were performed. As shown in Figure 4, no cytotoxic effect was shown for any of the tested solutions, with no statistical differences. Three independent experiments were performed. Then, due to the best performance of the solution containing a 400 nM concentration of CrCP, we used this concentration for all subsequent functional assays on the HuDe cell line.

### 2.3. Effects of CrCP on HuDe Cell Motility

**Figure 5:**
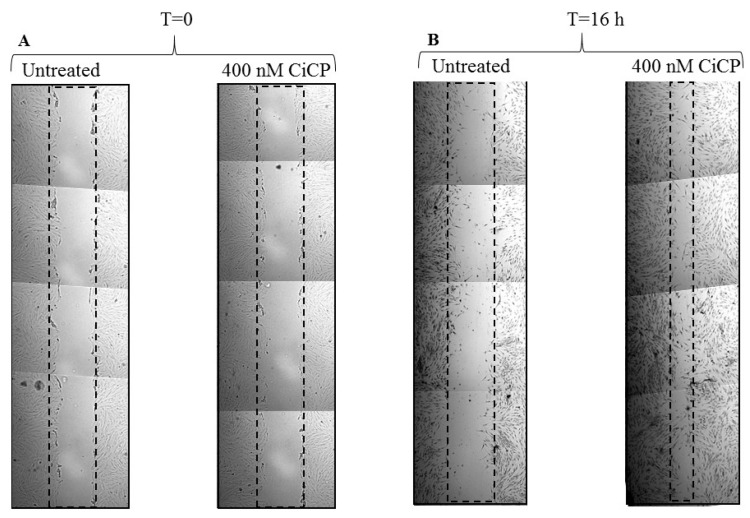
Wound healing assay: HuDe cell monolayers were scratched in a straight line with a pipette tip (**A**, T = 0) and then treated or not with a solution containing 400 nM of CrCP. The microscope observation shows an increment of cell migration in the sample treated with CrCP after 16 h of incubation (**B**, T = 16 h); no difference was observed in untreated wells (*p* = 0.003).

CrCP was used in cell motility assays to evaluate its biological activity. First of all, HuDe cells were scratched and incubated for 16 h with a solution containing 400 nM of CrCP. Then, the treated HuDe cells were observed under microscope magnification and compared to untreated control cells. In all the observations, a clear phenomenon of wound repair with increased cell migration was observed (Figure 5A,B). Three independent wound healing experiments were performed (*p* = 0.003).

**Figure 6:**
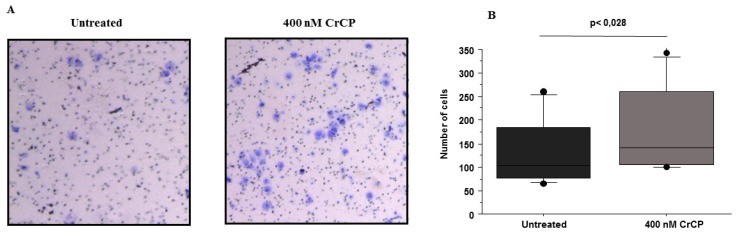
Transwell assay: Panel **A** shows microscope magnification of stained HuDe cells treated with a solution containing 400 nM of CrCP versus untreated cells. Panel **B** shows a box plot with the quantification of the cells migrating towards the CrCP-containing medium (*p* < 0.028). Statistical analysis was performed by a Wilcoxon signed-rank test.

Furthermore, a transwell migration assay was carried out to investigate the chemo-attracting ability of CrCP. For this reason, HuDe cells were seeded in the upper side of a Boyden chamber with a microporous membrane and, on the other side of the compartment, with culture medium supplemented with 400 nM of CrCP. After 16 h of treatment, cells were fixed, stained and counted under the microscope (Figure 6A). A statistical analysis of three independent experiments was performed and the data were analyzed by Wilcoxon signed-rank test (Figure 6B). The data obtained demonstrated that, in the presence of CrCP, a significant increase in HuDe cell motility was observed, demonstrating the chemo-attractive activity of the peptide.

### 2.4. CrCP Modulates the Expression of Cell Motility Genes

**Figure 7:**
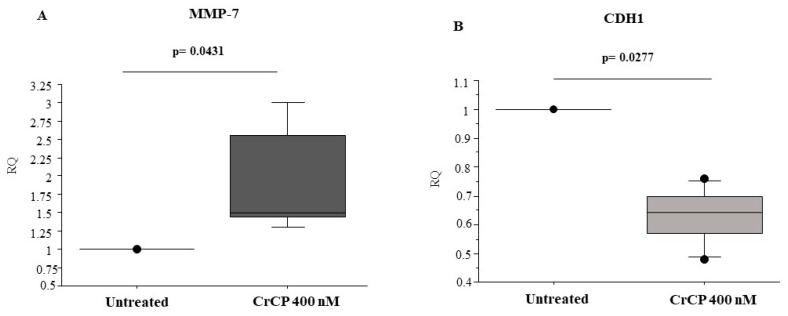
Cell motility gene expression: Real-Time PCR analysis displays a significant increase in MMP-7 (*p* = 0.0431) and a decrease in CDH1 (*p* = 0.0277) mRNA expression in HuDe cells after 16 h of treatment with a solution containing 400 nM of CrCP (Mann-Whitney *U* test).

To verify whether CrCP modulates the expression of genes involved in cell motility pathways, Real-Time PCR analysis was performed on cDNA prepared from HuDe cells treated with a solution containing 400 nM of the peptide for 16 h. Statistical analysis performed on CrCP-treated versus untreated HuDe cells demonstrates that CrCP induces a significant increase in matrix metalloproteinase-7 (MMP-7) (*p* = 0.0431) mRNA expression and a significant reduction in E-cadherin (CDH1) levels (*p* = 0.0277) compared to control cells (Figure 7 panels A and B, respectively). Other MMP genes, such as MMP-9 and MMP-12, were analyzed, and no differences were found in their mRNA expression (data not shown).

### 2.5. Western Blot Analysis

**Figure 8:**
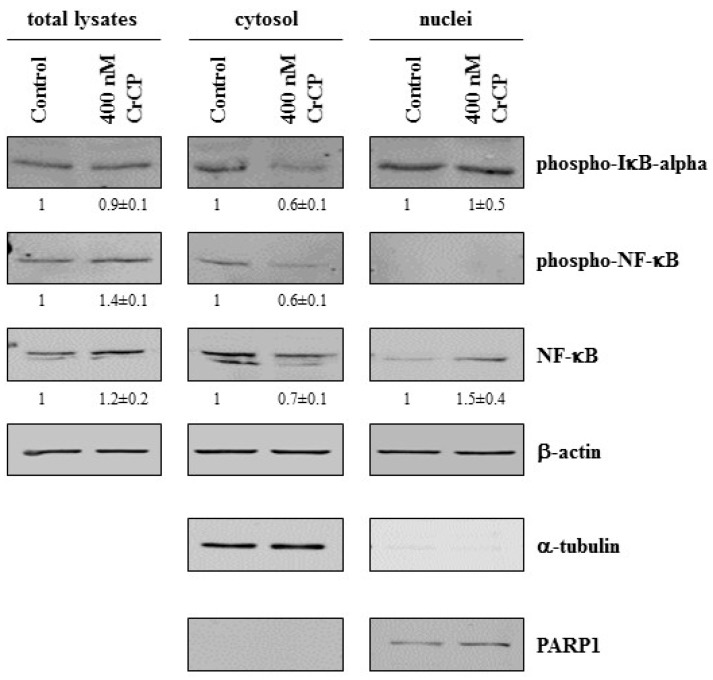
NF-κB translocates to the nucleus from the cytoplasm in HuDe cells after exposure to CrCP: western blot analysis of total, cytoplasmic and nuclear proteins from cells treated for two hours with the indicated concentration of CrCP. To evaluate the purity of the cytoplasmic and nuclear protein samples, antibodies to α-tubulin and PARP1 were used, respectively. Numbers indicate relative quantification ± SD after three independent experiments.

Several reports have shown that the transcription factor NF-κB is involved in the regulation of genes controlling cell migration, including MMPs and E-cadherin [34]. Therefore, to study the potential mechanism(s) responsible for the effects of CrCP on cellular behavior, HuDe cells were incubated with the peptide in serum-free medium for 2 h, and the expression levels of proteins involved in the NF-κB signaling pathway were analyzed. Under normal conditions, NF-κB interacts with IkB, and this interaction inhibits the nuclear translocation of NF-κB. In the presence of various types of signals, NF-κB translocates into the nucleus because of the phosphorylation, and subsequent degradation, of the inhibitor regulator IkB. To evaluate the alterations of NF-κB and IkB expression upon CrCP treatment, cells were exposed to a solution containing 400 nM of CrCP, and subcellular fractions were assayed by western blot (Figure 8). After incubation with CrCP, HuDe cells showed decreased expression of phospho-IkBα, total and phospho-NF-κB levels in the cytosol and increased expression levels of NF-κB in the nucleus. Collectively, these data suggest that treatment with CrCP induced the activation of the NF-κB signaling pathway.

## 3. Discussion

An inflammatory response induced by an external injury is characterized by a series of immediate events, such as hemostasis and inflammation, in which several factors are produced to recruit cells to the wound site [35]. The tissue infiltration of several cell types mediates essential processes for the resolution of the inflammatory process fighting pathogenic organisms, removing damaged tissue and apoptotic/necrotic cells, producing growth factors and promoting extracellular matrix (ECM) remodeling. This well-orchestrated series of events are the physiological pathways induced during an immune response.

Our research group published the identification of an LPS-induced CR-APA event leading to the upregulation of a truncated form of a longer mRNA (Ci8) with 3′ UTR cis-regulatory elements which have been demonstrated to be essential for innate immune response (C8short) [29]. Furthermore, we observed that the level of gene expression and tissue localization of the C8short mRNA were modulated by LPS injection, suggesting its involvement in the inflammatory immune response [29].

The C8short-derived peptide (CrCP) is a 73 aa-long peptide with no relevant homologies at the amino acid level, but with a peculiar amino acid composition containing a high percentage of prolines (22%) and glycine (13.7%) and having a pI of 4.00. So far, no data about its biological role in innate immune response has been postulated, though proline-rich motifs have been observed in many facets of the immune response, including antigen recognition, cell–cell communication and signal transduction and cellular reorganization events, which are essential for cell proliferation, secretion and migration [36].

To address this question, firstly we decided to perform molecular modeling studies in an attempt to hypothesize the three-dimensional folding of the peptide. The homology matching indicated that CrCP is structurally similar to a short domain (amino acids 192–240) of the vertebrate CRK gene (a homologue of the oncogene product of avian sarcoma virus, CT10 Regulator of Kinase) [31] with a special reference to a short stretch of proline and glycine residues (see Figure 1 for details). The vertebrate CRK gene encodes two spliced isoforms that have previously been recognized as adaptor components in multi-protein complexes that regulate the transcription and cytoskeletal reorganization essential for cell growth and motility by linking tyrosine kinases to small G proteins [37,38]. Our modeling studies show that CrCP displays a three-dimensional structure similar to the CRK domain (amino acids 192–240) with characteristics already reported for IDPs (biased amino acid composition and low sequence complexity) [39], suggesting a possible role of CrCP in cell migration and motility (Figure 2).

In *Ciona robusta*, the first physical barrier against invaders is the tunic, an extracellular matrix that is constantly exposed to all kinds of threats and foreign agents. For this reason, when damage occurs, an innate immune response is promptly triggered to eliminate potentially pathogenic microbes and, later, to restore tissue functionality [14]. In particular, ascidians have a leathery, cartilaginous or gelatinous tunic that entirely covers the epidermis of the animal with tunic cells distributed in the matrix. The tunic is a unique tissue in metazoans, mainly of epidermal origin and resembling vertebrate connective tissue [40]. In *Ciona robusta*, a few days after LPS inoculum in the tunic, it is possible to observe a wound repair response characterized by the formation of a thick capsule at the injection site [9,10]. In a wound environment, one of the key events of re-epithelialization after tissue injury is the loss of cell–cell and cell–ECM contacts by the epithelial cells at the edge of the wounded tissue, which assume a migratory phenotype [41,42]. Once the cells begin to migrate, the epithelial cells behind the wound edge begin to proliferate, and this continues until a new epithelium covers the damaged tissue [42]. Some of the key events observed during re-epithelization is the downregulation of adherent junction proteins, such as E-cadherin, and the upregulation of proteins capable of degrading the ECM, such as MMPs [43].

Starting from our homology studies, and taking in account the specificity of the ascidian tunic, we decided to set up an in vitro experimental model using the HuDe cell line to investigate the involvement of CrCP in cell motility.

As a first resort, we decided to test the ability of CrCP to stimulate cell migration in a wound-healing assay where monolayer HuDe cells were scratched and treated with the peptide. Figure 5 shows that, 16 h post incubation, scratched cells treated with CrCP showed a clear increase in cell motility, enhancing the ability of the HuDe cells to reduce the gap introduced by the physical damage; meanwhile, no effect was observed in untreated cells (*p* = 0.003) (Figure 5 panels A and B). Our analysis was further confirmed by a transwell migration assay performed using a Boyden chamber, by which we were able to show that treatment with CrCP preferentially induces the HuDe cells to cross the membrane apparatus, suggesting the chemo-attractive activity of the peptide (*p* = 0.028) (Figure 6 panels A and B).

Wound healing requires the controlled activity of several genes, including MMPs, whose role at all stages of the process has been clearly demonstrated [44]. The loss of MMP regulation is a characteristic of chronic wounds and contributes to the failure to heal. MMPs can orchestrate many additional inflammatory functions, such as regulating the migration of immune cells from the vasculature to the site of inflammation [45]. In addition, they are also able to regulate the recruitment of other cell types to the inflammation site by processing the ECM and regulating the bioavailability of growth factors, cytokines and chemokines [46]. For these reasons, by means of Real-Time PCR, gene expression studies were performed, looking at the pattern of expression of some MMPs (MMP-7, MMP-9, MMP-12) and E-cadherin genes after CrCP stimulation of HuDe cells.

MMP-7 is a matrilysin with a significant role in injured lung epithelium and is required for re-epithelialization after wounding of tracheal explants [47]. In fact, in the absence of MMP-7, re-epithelialization of tracheal wounds is almost completely abrogated [48], and this biological role can be extended to many other mucosal epithelia, in stomach and intestinal ulcers, injured epithelial cells in the kidney and basal epithelial cells during corneal wound healing [49]. Moreover, MMP-7 regulates re-epithelialization by cleavage of E-cadherin within the adherents’ junction, which facilitates the migration of epithelial cells away from the wound edge [48]. On the other hand, cadherins belong to the superfamily of cell-adhesion molecules (CAMs) [50] characterized by their adhesion properties mediated through repeated extracellular domains, playing a key role in cell-to-cell interactions. In our assay, we showed that treatment with CrCP statistically increased the expression of MMP-7 while decreasing E-cadherin gene expression, probably interfering with the production of junctional proteins inducing a migratory phenotype (Figure 7) [43].

The nuclear factor NF-κB is a well-established master regulator of inflammation and immunity [51] regulating a diverse range of cellular responses, among them proliferation, differentiation, programmed cell death and tumorigenesis. The NF-κB complex is usually inactive and located in the cytoplasm bound to its negative regulator, phospho-IκBα. Upon stimulation, NF-κB is released by the phosphorylation of its negative regulator and translated to the nucleus, activating several genes involved in immune response. In particular, during wound healing, the NF-κB pathway is usually activated in almost all cells, especially in macrophages and epithelial cells, making this event essential for the migration of phagocytic and inflammatory cells to the tissue [34]. Furthermore, this transcription factor also controls the expression of MMPs modulating the secretion and stability of several inflammatory cytokines and growth factors, which are essential for epidermal wound healing [52].

By means of western blotting analysis performed on CrCP-treated HuDe cells, we studied the potential intracellular molecular mechanisms induced by the peptide showing the activation of NF-κB signaling, as shown by the increase in NF-κB protein in the nuclei of the treated cells and the decrease in its negative regulator, phospho-IkBα, demonstrating that CrCP is able to activate NF-κB signaling (Figure 8).

Several reports have described the way that the immune response induced by foreign substances in the pharynx of the ascidian *Ciona robusta* promptly induces several responses, including phagocytosis, degranulation, enhanced humoral opsonizing lectins and the induction of inflammatory cytokines, which are followed by the formation of a capsule representing a reparative process [14]. In our study, we were able to show some evidence regarding the characterization of the biological ability of a synthetic peptide (CrCP) derived by the sequence of an LPS-induced mRNA upregulated during the inflammatory response in *Ciona robusta*. Such a peptide showed the ability to induce cell migration in a primary human dermal cell line via an NF-κB-dependent mechanism and leading to the modulation of the expression of relevant genes involved in cell motility. In this scenario, we could speculate that CrCP may be involved in re-epithelialization, restoring tissue architectures, probably through the loss of cell–cell contact, and allowing a migratory phenotype, suggesting that such an event may be involved in the resolution of the LPS-induced inflammatory reaction in *Ciona robusta*.

## 4. Material and Methods

### 4.1. Homology Modeling

The 3D structure of CrCP was constructed by homology modeling performed using PRIME software (PRIME, 2012). The sequence of the protein was retrieved from [29] and used as a FASTA sequence. The Basic Local Alignment performed by the Search Tool (BLAST) predicted the crystal structure of the CT10-Regulated Kinase isoform II (PDB ID: 2EYZ) as the most suitable average structure. The generated model was relaxed by molecular dynamics simulations and was further validated.

### 4.2. Molecular Dynamic Simulation

The MD simulation was carried out using the Desmond simulation package of Schrödinger LLC (Schrödinger Release 2017-3: Desmond Molecular Dynamics System; D. E. Shaw Research: New York, NY, 2017; Maestro-Desmond Interoperability Tools, Schrödinger, New York, NY, 2017). The NPT ensemble with a temperature of 300 K and a pressure of 1 bar was applied. The molecular system was neutralized by adding chloride. The volume of space in which the simulation took place (the global cell) was 90 Å × 90 Å × 90 Å. The molecular systems were solvated by SPC (simple point charge). The simulation run was 100 ns with a relaxation time of 1 ps. [53]. The trajectories were saved at 4.8 ps intervals for analysis. Behavior and interactions were analyzed using the Simulation Interaction Diagram tool implemented in the Desmond MD package. The Root Mean Square Deviations (RMSD) from the initial structure and the Root Mean Square Fluctuations (RMSF) were calculated during the simulation. All atoms were included in calculations of the RMSF.

### 4.3. Cell Culture and Materials

HuDe cells (BS PRC 41) were purchased from the Istituto Zooprofilattico Sperimentale of Lombardy and Emilia Romagna (Brescia, Italy) and maintained in culture with DMEM medium (Gibco, Life Technologies), supplemented with heat inactivated 10% Fetal Bovine Serum (FBS, Gibco, Life Technologies) and 1% antibiotic (penicillin 100 U/mL, Streptomycin sulfate 100 mg/mL, Invitrogen).

CrCP (NH2-MTSTVAIPQFFGNYPGVIPGSVPGGIPCPIPGTMPPANVPIPTSANGVSYPTVPIQVPIQLPVVPVGGGCYNE-COOH) was synthesized by Selleck Chemicals and was resuspended at a concentration of 10 μg/μL in highly purified distilled water (Lonza, BioWhittaker, USA).

### 4.4. MTS Assay

Cell viability was determined in vitro by 3-(4,5-dimethylthiazol-2-yl)-5-(3-carboxymethoxyphenyl)-2-(4-sulfophenyl)-2H-tetrazolium) MTS assay, using the CellTiter 96^®^ Aqueous One Solution Cell Proliferation Assay kit (Promega, USA), according to the manufacturer’s protocol. HuDe cells were seeded at a concentration of 3 × 10^3^ in 96-well tissue culture plates and incubated at 37 °C and 5% CO_2_ for 24 h. Then, cells were treated with increasing concentrations of CrCP (200 nM, 400 nM, 800 nM, 1.6 µM and 3.2 µM) and incubated at 37 °C and 5% CO_2_ for 24 h. At the defined time point, 20 μL of MTS solution were added and cells were further incubated in the same conditions for an additional 4 h. The absorbance of the dissolved formazan was measured in an automated microplate reader (Imark Plate Reader-BioRad) at 490 nm. Cell viability percentage was determined as the ratio between the absorbances (OD) of treated versus untreated cells × 100.

### 4.5. Wound-Healing Assay

The HuDe cells were seeded in six-well plates. When 70% cell confluence was reached, monolayer cells were scratched in a straight line with a pipet tip; the cell debris and suspended cells were removed by washing the cells with serum-free DMEM. Images were then captured by aligning the dishes with the reference point made at 0 h using a phase contrast microscope (LEICA, Milan, Italy). The cells were incubated in complete DMEM medium (10% FBS) and treated with or without CrCP (400 nM) for 16 h at 37 °C and 5% CO_2_. After incubation, cells were washed twice in 1 × PBS (Ca^2+^ and Mg^2+)^ (cat. number ECB4053L EUROCLONE, Milan, Italy) and fixed with 70% ethanol for 20 min at 4 °C. Two washes in 1 × PBS (Ca^2+^ and Mg^2+^) were carried out, and then cells were stained with 0.1% Crystal Violet solution for 5 min at room temperature. The dye residues were removed by washing with UltraPureTM distilled water DNAse/RNAse free (Invitrogen™, Thermo Fisher Scientific, Milan, Italy). Images were captured with a phase contrast microscope (LEICA, Milan, Italy) to evaluate cell migration. Experiments were performed in triplicate. For statistical analysis, ten overlapping shots were taken for each petri dish using a phase contrast microscope. The distance between the edges of the scars were quantified, and statistical analysis was performed by means of Mann-Whitney *U* test.

### 4.6. Migration Assay

The migration assays were performed in apposite chambers for 24-well plates (Falcon^®^ 24-well TC-treated Cell Polystyrene Permeable Support Companion Plate and cell culture PET insert (8.0 μm pore size). The chambers were filled with 100 μL of serum-free medium and incubated for 10 min at 37 °C. Then, 200 μL of HuDe cells (1× 10^4^/mL in serum-free medium) were placed in the upper insert of the chamber, and the respective wells filled with 800 μL of culture medium supplemented with 10% serum and 400 nM CrCP. Cells were incubated at 37 °C and 5% CO_2_ for 16 h. Thereafter, the medium was removed from the wells and cells were washed twice with PBS with Ca^2+^ and Mg^2+^. After fixing by paraformaldehyde (3.7% in PBS with Ca^2+^ and Mg^2+^), cells were incubated at room temperature for 10 min and washed with PBS with Ca^2+^ and Mg^2+^. Cells were stained with Crystal Violet 0.1% for 5 min at room temperature, washed again, and the non-migrating cells (in the upper face of migration chambers) were scraped off with cotton swabs. The migrated cells were counted using a phase contrast microscope (LEICA, Milan, Italy), and statistical analysis was performed by Wilcoxon signed-rank test.

### 4.7. Western Blotting Analyses

Whole-cell extracts were obtained from 1.5 × 10^6^ cells using RIPA buffer (Cell Signaling Technologies Inc., Danvers, MA). For cytoplasmic and nuclear protein extraction, the Nuclear Extract kit (Active Motif, Carlsbad, CA, USA) was used, and extraction was carried out according to the manufacturer’s instructions. Western blotting analyses were performed as previously reported [54], with primary antibodies against β-actin (Sigma-Aldrich), NF-κB (Santa Cruz, Dallas, Texas, USA), α-tubulin, PARP1, phospho-IkBα and phospho-NF-κB (Cell Signaling Technologies). Membranes were scanned and analyzed using the Odyssey^®^ infrared imaging system (LI-COR Biosciences, Lincoln, NE, USA) and Odyssey 3.0 imaging software. Equal loading and quality of cytosolic and nuclear proteins were determined using α-tubulin and PARP1 expression levels, respectively. The relative protein expressions were normalized using the quantified level of β-actin expression for total lysates, α-tubulin for cytosolic extracts and PARP1 for nuclei extracts, as previously reported [54]. The data presented are representative of three separate experiments with similar results.

### 4.8. Total RNA Preparation and qPCR Analysis

The HuDe cell line was seeded at a concentration of 0.5 × 10^6^ cells in a six-well tissue culture; after 24 h of incubation at 37 °C and 5% CO_2_, cells were washed with PBS (Gibco, Life Technologies) and treated with 400 nM of CrCP for 4 h at 37 °C and 5% CO_2_. Total RNA was extracted using the RNeasy Mini kit (Qiagen) according to the manufacturer’s protocol. The cDNA synthesis was performed using the QuantiTect Reverse Transcription Kit (Qiagen, Milan, Italy) from 2.5 μg total RNA. The cDNA was diluted up to 100 μL, and Real-Time PCR analyses were performed by Applied Biosystems StepOnePlusTM Real-Time PCR System and SYBR Green technology. Each sample contained 1–100 ng of cDNA in 2× Fast SYBR Green Master mix (Thermo Fisher, Milan, Italy) and 200 nM of specific Quantitect primer assay (Qiagen) in a final volume of 20 μL. MMP-7, MMP-9, MMP-12 and E-cadherin (CDH1) mRNA expression were evaluated using β-actin as a reference housekeeping gene. The primers used are reported in Table 1. PCR conditions were 95 °C for 20 s, followed by 40 cycles of two-step PCR denaturation at 95 °C for 15 s and annealing/extension at 60 °C for 30 s. Assays were performed four times.

### 4.9. Statistical Analysis

A Wilcoxon signed-rank test and Mann–Whitney *U* test were used for evaluating data, and *p* values < 0.05 were considered significant.

## 5. Conclusions

Marine invertebrates produce a large number of unique and structurally diversified natural products which are an important source of molecules with a wide range of bioactivities, such as anticancer, antiviral, antifungal and antibacterial properties. In this manuscript, we have characterized the chemo-attractive activity of CrCP, which is probably involved in the resolution of LPS-induced inflammatory response in the ascidian *Ciona robusta*.

## Figures and Tables

**Table 1:** Primers used in qPCR experiments.

Human Gene	Acronym	Genome Sequence	Code Number
β-actin	ACT	NM_001101	QT00095431
E-cadherin	CDH1	NM_004360	QT00080143
Matrix metalloprotease-7	MMP-7	NM_002423	QT00001456
Matrix metalloprotease-9	MMP-9	NM_004994	QT00040040
Matrix metalloprotease-12	MMP-12	NM_002426	QT01004472
